# Serum 25-hydroxyvitamin D levels and prevalent diabetic retinopathy in adults with type 2 diabetes: a retrospective hospital-based matched case-control study

**DOI:** 10.3389/fnut.2026.1828254

**Published:** 2026-10-07

**Authors:** Rong-Feng Dai, Yu Qian, Si-Qi Liu, Yu-Qing You, Yan Han, Yi Fang

**Affiliations:** Department of Endocrinology, Changzhou Third People’s Hospital, Changzhou, Jiangsu, China

**Keywords:** 25-hydroxyvitamin D, diabetic retinopathy, matched case–control study, type 2 diabetes mellitus, vitamin D deficiency

## Abstract

**Background:**

Diabetic retinopathy (DR) is a major microvascular complication of type 2 diabetes mellitus (T2DM). This study examined the adjusted association between serum 25-hydroxyvitamin D [25(OH)D] and prevalent DR.

**Methods:**

This retrospective, single-center matched case–control study was conducted at a tertiary hospital in China among adults with T2DM treated between January 2023 and March 2025. Serum 25(OH)D testing was not routine for all patients and was ordered at the treating clinician’s discretion. Participants were eligible only when a 25(OH)D result was available within ±7 days of bilateral fundus photography. Cases had prevalent DR and controls had no DR; individual 1:2 matching used sex, age within ±3 years, and diabetes duration within ±2 years. The prespecified primary analysis modeled 25(OH)D continuously in conditional logistic regression, and missing covariates were handled by multiple imputation.

**Results:**

The final sample included 399 selectively tested participants (133 with DR and 266 without DR) in 133 matched sets. Mean serum 25(OH)D was 18.6 ± 7.4 ng/mL in the DR group and 23.4 ± 8.1 ng/mL in the non-DR group. In the prespecified adjusted model, each 10 ng/mL higher serum 25(OH)D level was associated with lower prevalence odds of DR (OR, 0.62; 95% CI, 0.48–0.81; *p* < 0.001). Additional adjustment for continuous age and diabetes duration yielded a similar estimate (OR, 0.63; 95% CI, 0.48–0.83; *p* = 0.001). In an exploratory unadjusted analysis, mean 25(OH)D decreased across ICDR categories from no DR to proliferative DR (23.4 to 15.2 ng/mL; P for trend < 0.001).

**Conclusion:**

In this selectively tested, single-center tertiary-hospital sample, lower serum 25(OH)D was concurrently associated with higher prevalence odds of DR. Given the retrospective cross-sectional case–control design, this finding should be interpreted as an association only; it does not establish temporality or causality, and selection bias, residual confounding, and reverse causality remain possible.

## 1 Introduction

Diabetic retinopathy (DR) remains a leading microvascular complication of type 2 diabetes mellitus (T2DM) and a major cause of preventable vision impairment (1, 2). Contemporary clinical frameworks emphasize systematic retinal surveillance and timely referral because early-stage DR may be asymptomatic but remains amenable to risk-factor optimization and ophthalmic intervention (3, 4). Digital fundus photography is an effective approach for DR detection in appropriate settings (5, 6). Beyond established correlates such as glycemic exposure, blood pressure, lipid abnormalities, and renal impairment (4–7), serum 25-hydroxyvitamin D [25(OH)D] has been proposed as a marker related to diabetic microvascular health. Inflammatory, oxidative, endothelial, angiogenic, and vitamin D receptor pathways provide biological plausibility (8, 9), but none was measured in the present study and none constitutes mechanistic evidence for the observed association.

Previous evidence should be separated by design because different designs answer different questions. Cross-sectional and case–control studies estimate associations with prevalent DR but cannot establish temporality (10–14). Prospective evidence on incident DR or longitudinal progression is more limited and heterogeneous, while retinal imaging studies relate 25(OH)D to concurrent microvascular features rather than clinical incidence (14). Meta-analyses pool predominantly observational studies and therefore inherit between-study heterogeneity, residual confounding, and reverse-causality concerns (15–17). Mendelian randomization analyses have reported null or inconsistent evidence for a causal effect of genetically proxied 25(OH)D on DR or ocular outcomes (18, 19). Vitamin D supplementation studies were not designed to establish prevention or treatment of DR, and current evidence does not support a DR-specific supplementation effect (20, 21).

Against this background, before multivariable regression modeling, we specified the directional hypothesis that lower serum 25(OH)D would be associated with higher prevalence odds of DR. The study adds individual matching, a narrow exposure-outcome measurement interval, masked bilateral photographic grading, assay quality-control reporting, and alternative adjustment for selected renal and inflammatory variables. These features improve measurement and reporting quality but do not eliminate healthcare selection bias, control selection bias, residual confounding, or reverse causality. We therefore evaluated an adjusted association with prevalent DR rather than incident disease, progression, or causal effect.

## 2 Methods

### 2.1 Study design

This retrospective, single-center matched case–control study was conducted at Changzhou Third People’s Hospital, a tertiary Grade A hospital in Changzhou, China. The source population comprised consecutive adults with established T2DM who received outpatient or inpatient care between January 2023 and March 2025. Patients were not prospectively recruited. The analytical population was selective: participants had to undergo bilateral fundus photography and have a serum 25(OH)D measurement within ±7 days. Serum 25(OH)D testing was not routine for all adults with T2DM and was ordered at the treating clinician’s discretion, commonly during metabolic, nutritional, renal, or bone-health evaluation.

T2DM classification required a physician-recorded T2DM diagnosis and either (i) ICD-10 E11.x documentation on at least two encounters or (ii) antidiabetic treatment together with laboratory evidence meeting ADA diabetes criteria. Records with possible type 1, gestational, pancreatogenic, steroid-induced, monogenic, or other specific diabetes were independently reviewed by two endocrinologists and resolved by consensus. Eligibility additionally required age ≥18 years, gradable bilateral photography, complete matching variables, and an eligible serum 25(OH)D measurement within ±7 days.

Exclusion criteria were non-T2DM or another specific diabetes type; pregnancy; acute infection requiring systemic therapy within 4 weeks; major surgery or severe trauma within 3 months; documented regular vitamin D or calcium-vitamin D supplementation within 3 months; primary hyperparathyroidism, hypoparathyroidism, persistent clinically significant hypercalcemia or hypocalcemia, or diagnosed metabolic bone disease; active malignancy under treatment or untreated/recurrent within 5 years, except non-melanoma skin cancer; systemic inflammatory or autoimmune disease requiring glucocorticoid, immunosuppressive, or biologic therapy; medications affecting vitamin D metabolism within 3 months (systemic glucocorticoids, phenytoin, carbamazepine, phenobarbital, rifampicin, selected antiretroviral therapy, ketoconazole, cholestyramine, or orlistat); media opacity, retinal vein occlusion, advanced age-related macular degeneration, or another retinal disorder preventing DR grading; prior retinal photocoagulation or intravitreal anti-vascular endothelial growth factor therapy; and incomplete key data. Incomplete key data were defined as missing information that prevented confirmation of eligibility, determination of DR status or exposure timing, or completion of the matching variables. Missing non-matching covariates did not trigger exclusion and were handled by multiple imputation.

The study was reported according to STROBE (22). The study size was determined by all available eligible DR cases and a prespecified 1:2 control ratio. A precision calculation based on 133 cases, 266 controls, an exposure SD of approximately 8 ng/mL, and an assumed within-set exposure correlation of 0.20 indicated an expected standard error of approximately 0.14 for the log OR per 10 ng/mL; for an anticipated OR of 0.70, the expected 95% CI was approximately 0.53–0.92. The protocol was approved by the Ethics Committee of Changzhou Third People’s Hospital. Retrospective record access, dataset creation, and statistical analysis began only after ethics approval.

### 2.2 Outcome definition and grouping

The index date was the first chronologically eligible bilateral fundus photography examination during the study period for which a serum 25(OH)D result was available within ±7 days. This prespecified first-eligible-examination rule prevented discretionary selection among multiple photography dates. Final DR status was determined from standardized bilateral color photographs; routine dilated examination was not substituted when gradable photographs were available.

Retinal imaging was performed after pharmacologic mydriasis using a TRC-NW400 digital retinal camera (Topcon Corporation, Tokyo, Japan). Two 45° fields per eye were obtained, centered on the macula and optic disk. Both eyes were graded, and participant-level severity followed the more severe eye using the International Clinical Diabetic Retinopathy (ICDR) scale. The primary outcome was any prevalent DR above no apparent DR. Because two-field photography can miss peripheral lesions compared with seven-field or ultrawide-field protocols, underascertainment of DR presence or severity was possible. Diabetic macular edema was not systematically evaluated because optical coherence tomography and stereoscopic macular assessment were not uniformly available; the outcome therefore represents photographic DR severity rather than the complete clinical spectrum of diabetic retinal disease.

Image quality was assessed before grading based on focus, illumination, field definition, and visibility of the optic disk, macula, major retinal vessels, and retinal lesions. Images with insufficient quality were retaken when possible. Participants with persistently ungradable images that precluded reliable classification of DR presence or ICDR severity were excluded.

All retinal images were independently graded by two board-certified ophthalmologists who were masked to serum 25(OH)D and laboratory data. Kappa statistics were calculated from the two independent ratings before adjudication. Binary classifications used unweighted Cohen κ, and ICDR severity used quadratic-weighted κ. Disagreements were then adjudicated by a third senior ophthalmologist to determine the final study classification.

Patients with T2DM and any DR were cases. Eligible controls had no DR lesions on fundus photography. Individual 1:2 matching used exact sex, age within ±3 years, and diabetes duration within ±2 years. When more than two controls met the calipers, the two closest in age and then diabetes duration were selected; random sampling was used only for exact ties. This deterministic rule prioritized local balance but could reduce representativeness of the full non-DR pool and may depend on the order in which cases are processed. We therefore compared selected controls with 170 eligible non-selected controls and treated possible control selection bias as a limitation.

### 2.3 Exposure assessment and 25(OH)D measurement

The primary exposure was fasting morning serum 25(OH)D measured within ±7 days of the index photography. If more than one measurement was available, the closest measurement was selected. Prescription and nursing medication-reconciliation records, admission histories, and documented patient-reported supplements were reviewed for vitamin D or calcium-vitamin D use. Documented regular vitamin D intake of at least 400 IU/day for at least 4 weeks during the preceding 3 months was exclusionary. Supplements obtained outside the hospital could be missed when not reported; undocumented use was treated as unknown rather than confirmed absence.

Serum 25(OH)D was measured in the central laboratory using the Abbott ARCHITECT 25-OH Vitamin D chemiluminescent microparticle immunoassay on the ARCHITECT i2000SR analyzer (Abbott Laboratories, Abbott Park, IL, United States). Calibration was standardized against NIST SRM 2972, with method comparison against isotope-dilution LC–MS/MS. The analytical measuring interval was 3.4–155.9 ng/mL. Multi-level internal quality-control materials were analyzed with each batch; within-run coefficients of variation ranged from 2.0 to 2.3% and between-run coefficients of variation from 3.0 to 3.6%. The central laboratory operated under ISO 15189-accredited quality procedures and participated in external proficiency testing. Additional assay details are provided in Supplementary Table S1.

Serum 25(OH)D was analyzed primarily as a continuous variable per 10 ng/mL. Historical categories (<20, 20–29, and ≥30 ng/mL) were used only for secondary descriptive analyses and were not interpreted as DR-specific diagnostic, screening, or treatment thresholds. Exploratory analyses used sample-based quartiles. Seasons were defined as spring (March–May), summer (June–August), autumn (September–November), and winter (December–February); month of collection was used in sensitivity analysis.

### 2.4 Covariates and medication variables

Covariates were extracted at or closest to the index date. Hypertension was defined by an ICD-10 I10–I15 diagnosis or clinician documentation plus antihypertensive treatment, or by two routine-care blood-pressure readings ≥140/90 mmHg. Dyslipidemia required an ICD-10 E78.x diagnosis, clinician documentation, or lipid-lowering treatment. Coronary artery disease required an ICD-10 I20–I25 diagnosis, documented myocardial infarction, coronary revascularization, or angiographically documented disease. Chronic kidney disease (CKD) required an ICD-10 N18.x diagnosis or eGFR <60 mL/min/1.73 m^2^ and/or UACR ≥30 mg/g documented for at least 3 months.

Blood pressure was measured in the seated position after at least 5 min of rest with a calibrated automated upper-arm device (Omron HBP-9020; Kyoto, Japan). Two readings were obtained at least 1 min apart, and their mean was used; when only one routine-care reading was documented, that value was retained and flagged in data cleaning. Laboratory samples were obtained within ±7 days. Serum creatinine was isotope-dilution mass-spectrometry-traceable, and eGFR was calculated with the 2021 CKD-EPI creatinine equation without a race coefficient (23). HbA1c, UACR, CRP, and albumin methods and standardization are detailed in Supplementary Table S2.

Medication variables were based on active institutional prescriptions or documented regular use at the index date. These included insulin, metformin, sulfonylurea or glinide, DPP-4 inhibitor, GLP-1 receptor agonist, SGLT2 inhibitor, antihypertensive drugs, renin–angiotensin system blockers, statins, and other lipid-lowering therapy. Absence of an active prescription or documented regular use was coded as no documented use, not confirmed non-use, because outside prescriptions and incomplete reconciliation could not be excluded.

### 2.5 Statistical analysis

All analyses used IBM SPSS Statistics, version 26.0 (IBM Corp., Armonk, NY, United States). The directional hypothesis and continuous exposure specification were defined before multivariable regression modeling. Continuous variables were summarized as mean ± SD or median (IQR), categorical variables as number (percentage), and group imbalance by conventional independent-group standardized mean differences (SMDs); SMDs did not account for matching and were interpreted descriptively. Conditional logistic regression stratified by matched set estimated prevalence odds ratios (ORs) and 95% CIs. The prespecified adjusted Model 1 included BMI, smoking, alcohol use, hypertension, dyslipidemia, coronary artery disease, and season; it was not claimed to be a proven sufficient causal adjustment set. All 133 matched sets were informative for the primary continuous exposure analysis.

The conceptual framework (Supplementary Figure S1) separates plausible background/shared factors from biomarker domains whose temporal and causal roles are uncertain. It is interpretive and is not presented as proof of a sufficient adjustment set. Age, sex, and diabetes duration were addressed by matching. Extended exploratory Model A added SBP, HbA1c, total cholesterol, eGFR, and log UACR; Extended exploratory Model B additionally added log CRP and albumin. These were alternative specifications rather than progressively more valid models because the added variables may be shared causes, disease-severity markers, mediators, or colliders. Model-complexity stability was examined by resampling matched sets with replacement in 1,000 bootstrap samples, leave-one-covariate-domain-out models, and leave-one-matched-set-out influence analysis. The leave-one-matched-set-out analysis was applied to Model 1 (Supplementary Table S3).

Missing covariates were handled by multiple imputation by chained equations with 20 datasets and 20 iterations. The imputation model included DR status, serum 25(OH)D, matching variables, season, available covariates, and medication variables, but not the 133-level matched-set identifier. Conditional logistic regression coefficients and standard errors from the imputed datasets were combined using Rubin’s rules. Serum creatinine was imputed first; eGFR was then recalculated passively with the 2021 CKD-EPI equation. CKD was recalculated within each imputed dataset from longitudinal CKD evidence, passive eGFR, and UACR rather than imputed independently. UACR, CRP, and triglycerides were log-transformed before imputation. The missing-data pattern and imputation handling are summarized in Supplementary Table S4. Convergence and observed-versus-imputed distributions are provided in Supplementary Figure S2 and Supplementary Table S5. Departures from missing at random were explored by multiplying imputed UACR and CRP values by 0.75, 1.25, or 1.50 (Supplementary Table S6).

Historical 25(OH)D categories, quartiles, cross-sectional ICDR severity, subgroups, medication/month specifications, and restricted cubic splines were secondary or exploratory. The categorical model contained 94 informative matched sets and the quartile model 108. Quartile trend assigned each participant the median 25(OH)D value of their quartile. The spline used four knots at the 5th, 35th, 65th, and 95th percentiles and the sample median (22.0 ng/mL) as reference; a rug plot displayed the exposure distribution. For subgroup estimates, only intact matched triads in which all three participants belonged to the same stratum were retained; interaction tests in the full matched sample were the primary basis for effect-modification assessment. ICDR severity patterns were analyzed descriptively with the Jonckheere–Terpstra test for continuous 25(OH)D and the Cochran–Armitage trend test for the proportion <20 ng/mL; these analyses were unadjusted and did not incorporate matching. Linearity of continuous covariates was assessed with restricted cubic splines, and influence was assessed by leave-one-matched-set-out changes in the 25(OH)D coefficient. No multiplicity correction was applied to secondary analyses.

## 3 Results

### 3.1 Study population and selection

During the study period, 2,864 consecutive adults with T2DM received care and 1,127 underwent fundus photography. Of these, 423 were not taken forward to detailed screening because no eligible serum 25(OH)D test was available within ±7 days (*n* = 361), linked clinical records were insufficient (*n* = 34), diabetes type was uncertain or not T2DM at preliminary review (*n* = 18), or the index photographs were unavailable/ungradable (*n* = 10). The remaining 704 entered detailed screening; 666 had a qualifying 25(OH)D result. A further 97 were excluded for non-T2DM/specific diabetes type (*n* = 4), unavailable/ungradable photographs (*n* = 26), exclusionary systemic or ocular conditions (*n* = 29), vitamin D supplementation or medication affecting vitamin D metabolism (*n* = 16), or incomplete key data (*n* = 22). This left 133 eligible DR cases and 436 eligible non-DR controls. Matching selected 266 controls; 170 eligible controls were not selected. The final sample comprised 399 participants in 133 matched sets (Figure 1).

**Figure 1:**
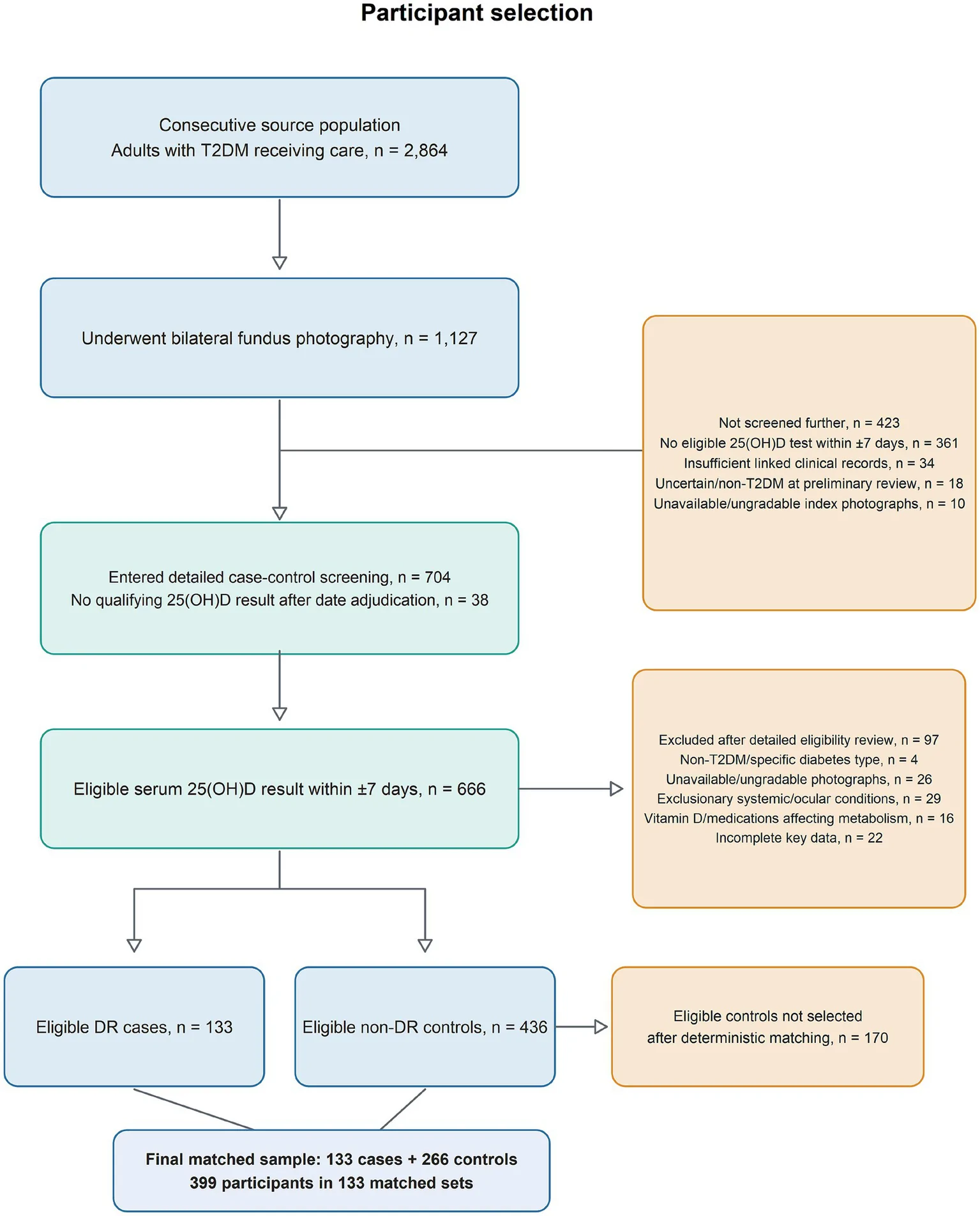
Participant selection from the consecutive source population to the final matched analytical sample. Among 704 patients entering detailed screening, 38 had no qualifying 25(OH)D result after date adjudication. Reasons for the 423 patients not entering detailed screening and for the 97 later exclusions are mutually exclusive within each stage.

### 3.2 Baseline characteristics

The final matched analytical sample included 133 participants with DR and 266 without DR. Matching addressed only sex, age within a caliper, and diabetes duration within a caliper; it did not produce broad clinical comparability. Conventional SMDs were 0.18 for age, 0.00 for sex, and 0.02 for diabetes duration, while substantial imbalances remained for SBP, HbA1c, renal measures, CRP, and medication use (Table 1). Compared with the 423 photographed patients not taken forward to detailed screening, the 704 patients entering detailed screening were older, more often inpatients, had longer diabetes duration, and had lower eGFR (Supplementary Table S7), supporting healthcare selection bias. Selected controls were younger and had shorter diabetes duration than 170 eligible non-selected controls, although their serum 25(OH)D distributions were similar (23.4 ± 8.1 vs. 22.6 ± 8.0 ng/mL; SMD = 0.10; Supplementary Table S8).

**Table 1:** Descriptive baseline characteristics of patients with T2DM by DR status.

Variable	DR (*n* = 133)	Non-DR (*n* = 266)	SMD
Age, years	61.1 ± 8.3	59.6 ± 8.5	0.18
Male sex, *n* (%)	80 (60.2)	160 (60.2)	0.00
BMI, kg/m^2^ (*n* = 131/260)	25.4 ± 3.6	25.3 ± 3.3	0.03
Smoking status, *n* (%)			0.13
Never	72 (54.1)	155 (58.3)	
Former	28 (21.1)	60 (22.6)	
Current	33 (24.8)	51 (19.2)	
Alcohol use, *n* (%)			0.07
Never	89 (66.9)	181 (68.0)	
Former	13 (9.8)	32 (12.0)	
Current	31 (23.3)	53 (19.9)	
Duration of diabetes, years	10.2 ± 5.2	10.1 ± 5.0	0.02
Season of blood collection, *n* (%)			0.17
Spring	35 (26.3)	67 (25.2)	
Summer	29 (21.8)	75 (28.2)	
Autumn	31 (23.3)	66 (24.8)	
Winter	38 (28.6)	58 (21.8)	
SBP, mmHg (*n* = 132/264)	138.9 ± 15.1	131.3 ± 15.4	0.50
DBP, mmHg (*n* = 132/264)	83.5 ± 9.0	79.9 ± 10.0	0.38
Hypertension, *n* (%)	92 (69.2)	163 (61.3)	0.17
Dyslipidemia, *n* (%)	78 (58.6)	144 (54.1)	0.09
Coronary artery disease, *n* (%)	23 (17.3)	30 (11.3)	0.17
Chronic kidney disease, *n* (%)	25 (18.8)	27 (10.2)	0.25
HbA1c, % (*n* = 130/259)	8.3 ± 1.3	7.7 ± 1.3	0.46
FPG, mmol/L (*n* = 129/258)	10.1 ± 2.7	9.0 ± 2.6	0.42
TC, mmol/L (*n* = 131/262)	5.03 ± 0.97	4.67 ± 0.80	0.40
TG, mmol/L (*n* = 130/262)	1.93 (1.42, 2.65)	1.72 (1.23, 2.30)	0.22
HDL-C, mmol/L (*n* = 131/263)	1.10 ± 0.26	1.10 ± 0.24	0.00
LDL-C, mmol/L (*n* = 131/262)	2.70 ± 0.79	2.61 ± 0.80	0.11
Scr, μmol/L (*n* = 132/263)	92 ± 22	87 ± 19	0.24
eGFR, mL/min/1.73 m^2^ (*n* = 132/263)	75.4 ± 19.0	86.7 ± 16.6	0.63
UACR, mg/g (*n* = 120/243)	42.8 (26.3, 82.0)	25.5 (14.5, 47.4)	0.55
CRP, mg/L (*n* = 126/251)	3.6 (2.2, 5.4)	2.3 (1.4, 3.7)	0.53
Albumin, g/L (*n* = 131/262)	41.2 ± 3.0	41.8 ± 3.1	0.20

Values are mean ± standard deviation, median (interquartile range), or number (percentage). Baseline characteristics were summarized descriptively; SMDs quantified group differences. Variable labels show observed denominators as DR/non-DR where data were missing. Serum 25(OH)D, DR status, matching variables, and season were complete. For skewed TG, UACR, and CRP, SMDs used log-transformed values.

T2DM, type 2 diabetes mellitus; DR, diabetic retinopathy; SMD, standardized mean difference; BMI, body mass index; SBP, systolic blood pressure; DBP, diastolic blood pressure; HbA1c, glycated hemoglobin; FPG, fasting plasma glucose; TC, total cholesterol; TG, triglycerides; HDL-C, high-density lipoprotein cholesterol; LDL-C, low-density lipoprotein cholesterol; Scr, serum creatinine; eGFR, estimated glomerular filtration rate; UACR, urine albumin-to-creatinine ratio; CRP, C-reactive protein.

### 3.3 Distribution of 25(OH)D

Serum 25(OH)D distributions and historical categories are shown descriptively in Table 2. Mean serum 25(OH)D was 18.6 ± 7.4 ng/mL in the DR group and 23.4 ± 8.1 ng/mL in the non-DR group. Unmatched *p*-values were removed because they did not account for the matched design; matched conditional models provide the inferential estimates.

**Table 2:** Distribution of serum 25(OH)D and historical 25(OH)D categories by DR status.

Variable	DR (*n* = 133)	Non-DR (*n* = 266)
Serum 25(OH)D, ng/mL	18.6 ± 7.4	23.4 ± 8.1
Historical 25(OH)D category, *n* (%)		
<20 ng/mL	86 (64.7)	121 (45.5)
20–29 ng/mL	33 (24.8)	88 (33.1)
≥30 ng/mL	14 (10.5)	57 (21.4)

Values are descriptive. Unmatched *p*-values were removed because they do not account for individual matching. Inferential matched analyses are presented in Tables 3, 4.

### 3.4 Inter-rater reliability for DR assessment

Pre-adjudication inter-rater reliability is presented in Supplementary Table S9. Agreement was 381/399 (95.5%) for DR presence (*κ* = 0.90; 95% CI, 0.86–0.94), 348/399 (87.2%) for ICDR severity (quadratic-weighted *κ* = 0.86; 95% CI, 0.82–0.91), and 370/399 (92.7%) for referable DR (*κ* = 0.84; 95% CI, 0.78–0.89).

### 3.5 Association between serum 25(OH)D and DR

Conditional logistic regression stratified by matched set evaluated the adjusted association between serum 25(OH)D and prevalent DR (Table 3). All 133 matched sets contributed information to the continuous analysis.

**Table 3:** Association between serum 25(OH)D and prevalent diabetic retinopathy: primary continuous analysis.

Model	*n*	Matched sets	Informative sets	OR per 10 ng/mL (95% CI)	*p*-value
Crude model	399	133	133	0.55 (0.43–0.70)	<0.001
Prespecified adjusted Model 1	399	133	133	0.62 (0.48–0.81)	<0.001
Extended exploratory Model A	399	133	133	0.66 (0.50–0.87)	0.003
Extended exploratory Model B	399	133	133	0.69 (0.52–0.91)	0.009

Odds ratios were estimated using conditional logistic regression stratified by matched set. The prespecified adjusted Model 1 included BMI, smoking, alcohol use, hypertension, dyslipidemia, coronary artery disease, and season. Extended exploratory Model A additionally included SBP, HbA1c, total cholesterol, eGFR, and log UACR; Extended exploratory Model B additionally included log CRP and albumin. The extended models were alternative exploratory specifications and were not interpreted as progressively more valid causal models.

The crude OR per 10 ng/mL higher serum 25(OH)D was 0.55 (95% CI, 0.43–0.70; *p* < 0.001). In the prespecified adjusted Model 1, the OR was 0.62 (95% CI, 0.48–0.81; *p* < 0.001). Estimates were 0.66 (95% CI, 0.50–0.87; *p* = 0.003) in extended exploratory Model A and 0.69 (95% CI, 0.52–0.91; *p* = 0.009) in extended exploratory Model B. This attenuation after additional covariate adjustment may reflect control of shared causes, adjustment for disease-severity markers, overadjustment, measurement error, or conditioning on mediators/colliders; it was not interpreted solely as removal of confounding.

Historical 25(OH)D categories were analyzed as a secondary exploratory specification using 94 informative matched sets (Table 4). The ≥30 ng/mL group contained only 14 DR cases, producing imprecise contrasts. These categories were not interpreted as DR-specific thresholds or as screening/treatment cutoffs, and primary inference remained based on the continuous model.

**Table 4:** Exploratory association between historical 25(OH)D categories and prevalent diabetic retinopathy.

Historical category	DR/Non-DR, *n*	Informative sets	Crude OR (95% CI)	*P*-value	Extended exploratory Model B OR (95% CI)	*P*-value
≥30 ng/mL	14/57	94	1.00 (ref)	–	1.00 (ref)	–
20–29 ng/mL	33/88	94	1.65 (0.86–3.16)	0.131	1.38 (0.70–2.72)	0.352
<20 ng/mL	86/121	94	2.75 (1.45–5.22)	0.002	2.20 (1.12–4.33)	0.022
*P* for trend	–	94	–	<0.001	–	0.006

The category model included 94 informative matched sets. The ≥30 ng/mL group was retained as a historical reference for descriptive comparability, but it contained only 14 DR cases. These categories are not DR-specific thresholds and should not be used as screening or treatment cutoffs.

### 3.6 Serum 25(OH)D across ICDR severity categories

Serum 25(OH)D showed a concurrent, unadjusted gradient across ICDR categories (Supplementary Table S10; Jonckheere–Terpstra *p* < 0.001 for continuous 25(OH)D; Cochran–Armitage *p* = 0.002 for <20 ng/mL). Mean 25(OH)D was 23.4 ± 8.1 ng/mL in participants with no apparent DR, 20.5 ± 7.2 ng/mL in mild NPDR, 18.7 ± 7.0 ng/mL in moderate NPDR, 16.9 ± 6.5 ng/mL in severe NPDR, and 15.2 ± 6.0 ng/mL in PDR; the corresponding proportions with 25(OH)D < 20 ng/mL were 45.5, 55.6, 63.3, 78.3, and 75.0%, respectively. Because severity and exposure were measured at the same index assessment and matching was not incorporated into this descriptive analysis, the pattern does not establish onset, progression, or a causal contribution of low 25(OH)D to worsening DR.

### 3.7 Exploratory subgroup analyses

Exploratory subgroup estimates retained only intact matched triads whose members were all within the same stratum (Table 5); discordant or missing sets were excluded from stratum-specific models. Interaction tests in the full matched sample were the primary evidence. No interaction was statistically significant, but non-significant interaction *p*-values do not establish uniformity, especially given limited informative sets. We therefore avoid describing the association as consistent across all subgroups.

**Table 5:** Exploratory subgroup analyses for the association between serum 25(OH)D and diabetic retinopathy.

Subgroup	Category	*n*	Intact informative matched sets	OR per 10 ng/mL (95% CI)	*P*-value	*P* for interaction
HbA1c	<7.0%	87	29	0.74 (0.48–1.14)	0.168	0.512
	≥7.0%	243	81	0.65 (0.47–0.90)	0.009	
Diabetes duration	<10 years	174	58	0.68 (0.47–0.99)	0.044	0.906
	≥10 years	201	67	0.69 (0.49–0.98)	0.038	
BMI	<25 kg/m^2^	144	48	0.73 (0.49–1.09)	0.121	0.735
	≥25 kg/m^2^	201	67	0.66 (0.47–0.93)	0.017	
eGFR	≥60 mL/min/1.73 m^2^	306	102	0.68 (0.50–0.92)	0.012	0.587
	<60 mL/min/1.73 m^2^	33	11	0.79 (0.43–1.45)	0.447	
Sex	Female	159	53	0.71 (0.50–1.01)	0.058	0.640
	Male	240	80	0.67 (0.49–0.91)	0.011	

Stratum-specific estimates retained only intact 1:2 matched triads whose three members were in the same subgroup category; discordant or missing sets were excluded. Interaction terms in the full matched sample were the primary basis for effect-modification assessment. Non-significant interaction p values do not establish uniformity across subgroups. Analyses were exploratory and were not multiplicity-adjusted.

25(OH)D, 25-hydroxyvitamin D; OR, odds ratio; CI, confidence interval; HbA1c, glycated hemoglobin; BMI, body mass index; eGFR, estimated glomerular filtration rate.

### 3.8 Missing data handling and sensitivity analyses

Missingness was greatest for UACR (36/399, 9.0%) and CRP (22/399, 5.5%). Serum creatinine was imputed before passive eGFR calculation, and CKD was recalculated rather than independently imputed. Representative chain traces stabilized after approximately 10 iterations, and observed-versus-imputed distributions were similar (Supplementary Figure S2 and Supplementary Table S5). The missing-at-random assumption remains uncertain because UACR and CRP may have been ordered preferentially in participants with suspected renal disease, inflammation, or greater illness severity.

Sensitivity analyses are presented in Table 6 and Supplementary Tables S3, S5, S6. Additional adjustment for continuous age and diabetes duration produced an OR of 0.63 (95% CI, 0.48–0.83; *p* = 0.001). The matched-set bootstrap estimate for extended exploratory Model B was 0.70 (95% percentile interval, 0.50–0.96), and leave-one-covariate-domain-out estimates ranged from 0.64 to 0.66. In the Model 1 leave-one-matched-set-out analysis, estimates ranged from 0.60 to 0.64. Delta adjustments for potentially higher or lower missing UACR/CRP values yielded ORs of 0.69–0.70. These analyses indicate limited numerical sensitivity but do not remove selection bias or unmeasured confounding.

**Table 6:** Extended and sensitivity analyses for serum 25(OH)D and prevalent diabetic retinopathy.

Analysis	*n* (DR/non-DR)	Matched sets	OR per 10 ng/mL (95% CI)	*p*-value
Extended exploratory Model B, multiply imputed	399 (133/266)	133	0.69 (0.52–0.91)	0.009
Model 1 plus continuous age and diabetes duration	399 (133/266)	133	0.63 (0.48–0.83)	0.001
Complete-case intact 1:2 sets	336 (112/224)	112	0.70 (0.53–0.93)	0.014
Matched-set bootstrap, Extended exploratory Model B	399 (133/266)	133	0.70 (0.50–0.96)*	–
MNAR delta: missing UACR/CRP × 1.50	399 (133/266)	133	0.70 (0.53–0.93)	0.012

Odds ratios were estimated using conditional logistic regression stratified by matched set. *The bootstrap interval is the 2.5th–97.5th percentile interval from 1,000 matched-set bootstrap samples. MNAR denotes missing not at random. Additional model-stability and delta-sensitivity results are provided in Supplementary Tables S3, S6.

Additional exploratory month- and medication-adjusted models are shown in Supplementary Table S11. Medication distributions by DR status are presented in Supplementary Table S12. Medication variables represented no documented use rather than confirmed non-use. Attenuation and wider intervals after medication adjustment may reflect treatment intensity, exposure misclassification, or model complexity and were not treated as confirmation.

Exploratory quartile analyses used 108 informative matched sets and a trend score based on quartile-specific median 25(OH)D values (Supplementary Table S13). Although the lowest quartile had the largest OR, quartile boundaries were sample-derived and the spline did not support non-linearity. The analysis therefore does not identify a threshold at 17 ng/mL or any other cutoff.

Restricted cubic spline analysis used the sample median (22.0 ng/mL) as reference and displayed the observed exposure distribution with a rug plot (Supplementary Figure S3). The overall association was statistically significant (*p* = 0.008), whereas non-linearity was not (*p* = 0.214). Functional-form checks for continuous covariates and leave-one-matched-set-out influence analysis did not materially alter the 25(OH)D estimate (Supplementary Table S3).

## 4 Discussion

In this retrospective, single-center matched case–control study, lower serum 25(OH)D was associated with higher prevalence odds of DR among selectively tested adults with T2DM at a tertiary hospital. Mean serum 25(OH)D was 4.8 ng/mL lower in participants with DR than in matched participants without DR. In the prespecified adjusted continuous model, each 10 ng/mL higher 25(OH)D concentration was associated with 38% lower prevalence odds of DR (OR, 0.62; 95% CI, 0.48–0.81). Additional adjustment for continuous age and diabetes duration yielded a similar estimate, whereas the association attenuated in extended exploratory models containing metabolic, renal, inflammatory, and nutritional variables. Historical categories, quartiles, cross-sectional severity, subgroup analyses, and the spline were secondary or exploratory. Taken together, the findings indicate a measurable concurrent association, but they do not establish that lower 25(OH)D preceded DR, contributed to its development, or would predict subsequent progression.

The DR is a clinically heterogeneous microvascular complication and an important cause of preventable visual impairment (1–3). Glycemic exposure, hypertension, dyslipidemia, renal dysfunction, and diabetes duration remain central determinants of DR occurrence and severity (4–6). Although matching achieved close balance for sex and diabetes duration and limited the age difference, it did not make the two groups broadly exchangeable. Participants with DR had higher blood pressure and HbA1c, less favorable renal measures, higher C-reactive protein (CRP), and different medication patterns. These imbalances may indicate greater systemic disease burden, treatment intensity, or healthcare contact. The attenuation from the crude estimate to the extended exploratory estimates is compatible with confounding by measured clinical characteristics, but it could also reflect measurement error, overadjustment, or conditioning on variables that are consequences of diabetes severity. Therefore, the adjusted estimates should not be interpreted as isolating a direct biological effect of vitamin D. Lower 25(OH)D may instead function as an integrated marker of metabolic health, renal function, nutrition, mobility, outdoor activity, frailty, or comorbidity among patients already receiving hospital-based diabetes care.

Several biological pathways could plausibly connect vitamin D status with diabetic retinal disease, including regulation of inflammatory signaling, oxidative stress, endothelial function, angiogenesis, and vitamin D receptor activity in ocular tissues (24, 25). However, the present study measured none of these pathways. Individual observational studies have generally reported lower circulating 25(OH)D concentrations or more frequent vitamin D deficiency among patients with prevalent DR (10–14). Reviews have consequently proposed that vitamin D-related immunomodulatory and vascular effects may be relevant to retinal microvascular injury (8, 9). These proposals remain hypotheses rather than mechanistic confirmation. CRP is a non-specific systemic inflammatory marker and cannot capture retinal inflammation, cytokine activity, oxidative injury, endothelial signaling, or local angiogenic processes. Similarly, eGFR and albuminuria may indicate shared microvascular disease burden rather than a renal pathway through which 25(OH)D influences DR; diabetic renal and retinal complications frequently coexist (7). The imprecise estimate in the low-eGFR subgroup and the absence of a statistically significant interaction provide no basis for claiming renal effect modification. Mechanistic interpretation should therefore remain subordinate to the epidemiological design and the possibility that low 25(OH)D reflects overall illness rather than a retinally active exposure.

The complementary exposure analyses do not establish a clinically meaningful threshold. The ≥30 ng/mL historical reference category contained only 14 DR cases, the quartile boundaries were sample-derived, and the spline test did not support non-linearity. Current vitamin D guidance uses concentration categories for broader skeletal or metabolic considerations rather than for DR diagnosis, screening, or treatment (20, 21). Meta-analyses and recent retrospective studies have reported an association between lower 25(OH)D and prevalent DR, but their estimates remain affected by heterogeneity in populations, assays, outcome grading, and covariate adjustment (15–17, 26). In contrast, Mendelian randomization studies have not provided consistent evidence that genetically proxied 25(OH)D causally influences DR or broader ocular outcomes (18, 19). This difference may reflect residual confounding, reverse causality, selection, population differences, or the distinction between lifelong genetically influenced exposure and a single clinical measurement. Accordingly, the present results do not support using 25(OH)D to predict DR, replace retinal screening, define a treatment cutoff, or recommend supplementation to prevent DR or slow its progression. Such claims require prospective prediction studies and randomized trials with retinal outcomes.

Several limitations define interpretation and generalizability. First, inclusion required two clinician-dependent investigations within a narrow interval. Patients entering detailed screening were older, more often inpatients, had longer diabetes duration, and had lower eGFR than photographed patients without eligible 25(OH)D data, supporting healthcare selection bias. Second, deterministic matching selected 266 of 436 eligible controls. Similar 25(OH)D distributions but differences in age and diabetes duration between selected and non-selected controls do not exclude control selection bias. Third, excluding previously treated retinal disease restricted the severity spectrum; two-field photography may miss peripheral lesions, and diabetic macular edema was not systematically assessed. Fourth, a single 25(OH)D measurement may not represent long-term exposure, and outside prescriptions or over-the-counter supplements may have been missed. Sunlight exposure, diet, activity, socioeconomic status, frailty, treatment adherence, and healthcare use were also unavailable. Multiple imputation and sensitivity analyses cannot eliminate missing-not-at-random mechanisms or unmeasured confounding. Finally, cross-sectional timing permits reverse causality because visual impairment, reduced mobility, renal disease, poor nutrition, or greater illness burden may lower 25(OH)D after DR develops. Future multicenter prospective studies should use population-based sampling, repeated exposure measurements, wider-field retinal assessment, and longitudinal incident or progression outcomes. Until then, these findings should be interpreted as hypothesis-generating rather than practice-changing.

## 5 Conclusion

This retrospective, single-center matched case–control study identified a concurrent inverse association between serum 25(OH)D and prevalent DR among selectively tested patients at a tertiary hospital. Because exposure and outcome were assessed at the same index evaluation, the findings should be interpreted as associational and hypothesis-generating; they do not establish incident risk, progression, temporality, causality, clinical prediction value, or benefit from vitamin D testing or supplementation. Healthcare selection bias, control selection bias, residual confounding, and reverse causality limit generalization to community and primary-care populations.

## Data Availability

The raw data supporting the conclusions of this article will be made available by the authors, without undue reservation.

## References

[1] Kropp M Golubnitschaja O Mazurakova A Koklesova L Sargheini N Vo TKS . Diabetic retinopathy as the leading cause of blindness and early predictor of cascading complications—risks and mitigation. EPMA J. (2023) 14:21–42. doi: 10.1007/s13167-023-00314-8, 36866156 PMC9971534

[2] Gettinger K Lee D Tomita Y Negishi K Kurihara T. Diabetic retinopathy, a comprehensive overview on pathophysiology and relevant experimental models. Int J Mol Sci. (2025) 26:9882. doi: 10.3390/ijms26209882, 41155177 PMC12564527

[3] Upadhyay T Prasad R Mathurkar S. A narrative review of the advances in screening methods for diabetic retinopathy: enhancing early detection and vision preservation. Cureus. (2024) 16:e53586. doi: 10.7759/cureus.53586, 38455792 PMC10918290

[4] American Diabetes Association Professional Practice Committee. 12. Retinopathy, neuropathy, and foot care: standards of care in diabetes—2024. Diabetes Care. (2024) 47:S231–43. doi: 10.2337/dc24-S012, 38078577 PMC10725803

[5] Cheung N Mitchell P Wong TY. Diabetic retinopathy. Lancet. (2010) 376:124–36. doi: 10.1016/S0140-6736(09)62124-3, 20580421

[6] Yau JWY Rogers SL Kawasaki R Lamoureux EL Kowalski JW Bek T . Global prevalence and major risk factors of diabetic retinopathy. Diabetes Care. (2012) 35:556–64. doi: 10.2337/dc11-1909, 22301125 PMC3322721

[7] Barrett EJ Liu Z Khamaisi M King GL Klein R Klein BEK . Diabetic microvascular disease: an Endocrine Society scientific statement. J Clin Endocrinol Metab. (2017) 102:4343–410. doi: 10.1210/jc.2017-01922, 29126250 PMC5718697

[8] Tecilazich F Formenti AM Giustina A. Role of vitamin D in diabetic retinopathy: pathophysiological and clinical aspects. Rev Endocr Metab Disord. (2021) 22:715–27. doi: 10.1007/s11154-020-09575-4, 33026598 PMC7538371

[9] Ruamviboonsuk V Grzybowski A. The roles of vitamins in diabetic retinopathy: a narrative review. J Clin Med. (2022) 11:6490. doi: 10.3390/jcm11216490, 36362717 PMC9656452

[10] Zhuang Y Zhuang Z Cai Q Hu X Huang H. Serum vitamin D is substantially reduced and predicts flares in diabetic retinopathy patients. J Diabetes Investig. (2024) 15:867–73. doi: 10.1111/jdi.14185, 38469994 PMC11215683

[11] Chen C Meng S Wu X Ciren W Shen J Zeding Z . Vitamin D deficiency and the risk of diabetic retinopathy in patients with type 2 diabetes in Tibet: a cross-sectional analysis. BMC Endocr Disord. (2024) 24:139. doi: 10.1186/s12902-024-01668-4, 39095726 PMC11295715

[12] Zahedi M Motahari MM Fakhri F Aphshari NM Poursharif S Jahed R . Is vitamin D deficiency associated with retinopathy in type 2 diabetes mellitus? A case-control study. Clin Nutr ESPEN. (2024) 59:158–61. doi: 10.1016/j.clnesp.2023.11.011, 38220370

[13] Patrick PA Visintainer PF Shi Q Weiss IA Brand DA. Vitamin D and retinopathy in adults with diabetes mellitus. Arch Ophthalmol. (2012) 130:756–60. doi: 10.1001/archophthalmol.2011.2749, 22801837

[14] Wei Z Wang K Liu Y Liu P Tang Y Chen L . Association between vitamin D and microvascular changes in early diabetic retinopathy in patients with type 2 diabetes. Diabetes Metab Syndr Obes. (2025) 18:4085–95. doi: 10.2147/DMSO.S562924, 41235316 PMC12607676

[15] Trott M Driscoll R Iraldo E Pardhan S. Associations between vitamin D status and sight-threatening and non-sight-threatening diabetic retinopathy: a systematic review and meta-analysis. J Diabetes Metab Disord. (2022) 21:1177–84. doi: 10.1007/s40200-022-01059-3, 35673423 PMC9167360

[16] Petrea CE Ghenciu LA Iacob R Stoicescu ER Săndesc D. Vitamin D deficiency as a risk factor for diabetic retinopathy: a systematic review and meta-analysis. Biomedicine. (2024) 13:68. doi: 10.3390/biomedicines13010068, 39857652 PMC11762121

[17] Yu G Cao C Shu X Yao L. Association between vitamin D deficiency and the risk of diabetic retinopathy in patients with type 2 diabetes: a meta-analysis. Mol Genet Genomic Med. (2026) 14:e70157. doi: 10.1002/mgg3.70157, 41454443 PMC12743042

[18] Huang C Luo D Sun M Fang G Wei M Zhang Y . No causal association between serum vitamin D levels and diabetic retinopathy: a Mendelian randomization analysis. Nutr Metab Cardiovasc Dis. (2024) 34:1295–304. doi: 10.1016/j.numecd.2024.01.033 38508994

[19] Luo X Ruan Z Liu L. Causal effect of the 25-hydroxyvitamin D concentration on ocular diseases: a Mendelian randomization study. Sci Rep. (2025) 15:8701. doi: 10.1038/s41598-025-93613-1, 40082667 PMC11906842

[20] Holick MF Binkley NC Bischoff-Ferrari HA Gordon CM Hanley DA Heaney RP . Evaluation, treatment, and prevention of vitamin D deficiency: an Endocrine Society clinical practice guideline. J Clin Endocrinol Metab. (2011) 96:1911–30. doi: 10.1210/jc.2011-0385, 21646368

[21] Demay MB Pittas AG Bikle DD Diab DL Kiely ME Lazaretti-Castro M . Vitamin D for the prevention of disease: an Endocrine Society clinical practice guideline. J Clin Endocrinol Metab. (2024) 109:1907–47. doi: 10.1210/clinem/dgae290, 38828931

[22] von Elm E Altman DG Egger M Pocock SJ Gøtzsche PC Vandenbroucke JP. The strengthening the reporting of observational studies in epidemiology (STROBE) statement: guidelines for reporting observational studies. J Clin Epidemiol. (2008) 61:344–9. doi: 10.1016/j.jclinepi.2007.11.008, 18313558

[23] Inker LA Eneanya ND Coresh J Tighiouart H Wang D Sang Y . New creatinine- and cystatin C-based equations to estimate GFR without race. N Engl J Med. (2021) 385:1737–49. doi: 10.1056/NEJMoa2102953, 34554658 PMC8822996

[24] Stoica AM Jurja S Dervis N. The role of vitamin D in retinal physiology. Rom J Ophthalmol. (2025) 69:315–21. doi: 10.22336/rjo.2025.51, 41189788 PMC12580662

[25] Mrugacz M Pieńczykowska K Bryl A. The role of vitamin D3 in ocular diseases. Nutrients. (2024) 16:1878. doi: 10.3390/nu16121878, 38931233 PMC11206323

[26] Hung KC Chang LC Chang YJ Ho CN Wu JY Liu WC . Vitamin D deficiency and diabetic retinopathy risk in patients with newly diagnosed type 2 diabetes mellitus: a retrospective analysis. Front Nutr. (2025) 12:1614287. doi: 10.3389/fnut.2025.1614287, 40521365 PMC12162301

