# Supplementary material for: Serum 25-hydroxyvitamin D levels and prevalent diabetic retinopathy in adults with type 2 diabetes: a retrospective hospital-based matched case-control study
Source: Front Nutr. 2026 Oct 7;13:1828254. doi: 10.3389/fnut.2026.1828254 (PMC13682978; doi:10.3389/fnut.2026.1828254)
Supplement: Supplementary file 1 [file Supplementary_file_1.DOCX]

Supplementary Table S1. Performance and laboratory quality-control information for serum 25(OH)D measurement

| Item | Description |
| --- | --- |
| Analyte | Serum 25-hydroxyvitamin D [25(OH)D] |
| Platform | Abbott ARCHITECT i2000SR analyzer |
| Measurement name | ARCHITECT 25-OH Vitamin D |
| Method | Chemiluminescent microparticle immunochemical method |
| Principle | Quantitative delayed one-step competitive method |
| Sample type used in this study | Fasting morning venous serum |
| Intended specimen types | Human serum and plasma |
| Calibration system | Six-level ARCHITECT 25-OH Vitamin D calibrator system |
| Calibrator concentrations | 0.0, 4.0, 10.0, 30.0, 75.0, and 160.0 ng/mL |
| Standardization | Standardized against NIST SRM 2972; method comparison performed against ID-LC-MS/MS |
| Internal QC materials | Multi-level ARCHITECT 25-OH Vitamin D control materials |
| QC target concentrations | Low control: 20.0 ng/mL; medium control: 40.0 ng/mL; high control: 75.0 ng/mL |
| Analytical measuring interval | 3.4–155.9 ng/mL |
| Linear range | 3.4–155.9 ng/mL |
| Limit of blank | 1.6 ng/mL |
| Limit of detection | 2.2 ng/mL |
| Limit of quantitation | 2.4 ng/mL at ≤20% CV |
| Low-level QC mean concentration | 20.6 ng/mL |
| Within-run CV, low-level QC | 2.10% |
| Between-run CV, low-level QC | 3.30% |
| Medium-level QC mean concentration | 40.6 ng/mL |
| Within-run CV, medium-level QC | 2.00% |
| Between-run CV, medium-level QC | 3.00% |
| High-level QC mean concentration | 77.3 ng/mL |
| Within-run CV, high-level QC | 2.30% |
| Between-run CV, high-level QC | 3.60% |
| Method comparison | Passing–Bablok regression slope 1.02, intercept −0.99, r = 0.99 versus ID-LC-MS/MS |
| Laboratory quality framework | ISO 15189-accredited procedures, batch-level internal quality control, and participation in external proficiency testing |

Supplementary Table S2. Laboratory methods for covariates used in adjusted models

| Analyte | Platform / method | Standardization / quality control |
| --- | --- | --- |
| Serum creatinine | Roche Cobas c702; enzymatic colorimetric assay | IDMS-traceable calibration; daily two-level internal QC |
| eGFR | Calculated from serum creatinine, age, and sex | 2021 CKD-EPI creatinine equation; no race coefficient |
| HbA1c | Tosoh HLC-723G8; high-performance liquid chromatography | NGSP-certified and IFCC-aligned; two-level daily QC |
| Urine albumin | Roche Cobas c702; immunoturbidimetry | Manufacturer calibrator; daily internal QC |
| Urine creatinine | Roche Cobas c702; enzymatic assay | IDMS-traceable; UACR calculated as mg/g |
| CRP | Roche Cobas c702; latex-enhanced immunoturbidimetry | Two-level daily internal QC |
| Serum albumin | Roche Cobas c702; bromocresol green method | Manufacturer calibration; daily internal QC |

Abbreviations: IDMS, isotope-dilution mass spectrometry; NGSP, National Glycohemoglobin Standardization Program; IFCC, International Federation of Clinical Chemistry; QC, quality control; UACR, urine albumin-to-creatinine ratio.

Supplementary Table S3. Model-complexity, functional-form, and influence stability analyses

| Analysis | OR per 10 ng/mL higher 25(OH)D (95% CI) | Interpretation |
| --- | --- | --- |
| Prespecified adjusted Model 1 | 0.62 (0.48–0.81) | Primary analysis |
| Model 1 + continuous age and diabetes duration | 0.63 (0.48–0.83) | Residual within-caliper adjustment |
| Extended exploratory Model B | 0.69 (0.52–0.91) | Alternative specification |
| Matched-set bootstrap (1,000 samples) | 0.70 (0.50–0.96)* | Accounts for model instability by resampling sets |
| Extended model without metabolic domain | 0.66 (0.50–0.87) | HbA1c/SBP/TC omitted |
| Extended model without renal domain | 0.64 (0.49–0.84) | eGFR/UACR omitted |
| Extended model without CRP/albumin | 0.66 (0.50–0.87) | Inflammatory/nutritional domain omitted |
| Leave-one-matched-set-out analysis, Model 1 | 0.60–0.64 | No single set changed the Model 1 log OR by >0.08 |
| Spline checks for SBP, HbA1c, TC, eGFR, albumin | All P for nonlinearity >0.10 | No strong departure from modeled linear form |

*2.5th–97.5th percentile interval. Leave-one-covariate-domain-out analyses used extended exploratory Model B; the leave-one-matched-set-out analysis used Model 1. These analyses evaluate numerical stability but do not prove absence of overadjustment, sparse-data bias, or residual confounding.

Supplementary Table S4. Missing data pattern and variables included in the imputation model

| Variable | Missing, n (%) | Imputation handling | Consistency rule |
| --- | --- | --- | --- |
| DR status | 0 | Predictor only | Not imputed |
| Serum 25(OH)D | 0 | Predictor only | Not imputed |
| Age / sex / diabetes duration | 0 | Predictors only | Not imputed |
| Season | 0 | Predictor only | Not imputed |
| BMI | 8 (2.0) | Predictive mean matching | — |
| HbA1c | 10 (2.5) | Predictive mean matching | — |
| FPG | 12 (3.0) | Predictive mean matching | — |
| Total cholesterol | 6 (1.5) | Predictive mean matching | — |
| Triglycerides | 7 (1.8) | Predictive mean matching after log transform | — |
| HDL-C | 5 (1.3) | Predictive mean matching | — |
| LDL-C | 6 (1.5) | Predictive mean matching | — |
| Serum creatinine | 4 (1.0) | Predictive mean matching | Imputed before eGFR |
| eGFR | 4 (1.0) | Passive recalculation | 2021 CKD-EPI equation |
| UACR | 36 (9.0) | Predictive mean matching after log transform | Used to recalculate CKD |
| CRP | 22 (5.5) | Predictive mean matching after log transform | — |
| Albumin | 6 (1.5) | Predictive mean matching | — |
| CKD | 0 | Recalculated in each dataset | Longitudinal CKD evidence + passive eGFR/UACR |
| Matched-set ID | 0 | Excluded from imputation predictors | Not represented by 133 indicator categories |

Notes: Twenty imputed datasets with 20 iterations were generated. The imputation model included outcome, exposure, matching variables, season, clinical/laboratory covariates, and medication variables, but not matched-set ID. Conditional logistic regression estimates were combined across imputed datasets using Rubin's rules. Serum creatinine was imputed first and eGFR was recalculated passively; CKD was recalculated rather than independently imputed. The missing-at-random assumption was evaluated with delta sensitivity analyses (Supplementary Table S6).

Supplementary Table S5. Observed and imputed distributions for representative incomplete variables

| Variable | Missing, n (%) | Observed distribution | Pooled imputed values |
| --- | --- | --- | --- |
| BMI, kg/m² | 8 (2.0) | 25.3 ± 3.4 | 25.4 ± 3.5 |
| HbA1c, % | 10 (2.5) | 7.9 ± 1.4 | 8.0 ± 1.3 |
| FPG, mmol/L | 12 (3.0) | 9.4 ± 2.7 | 9.5 ± 2.6 |
| UACR, mg/g | 36 (9.0) | 30.4 (16.2–59.8) | 31.0 (17.4–62.9) |
| CRP, mg/L | 22 (5.5) | 2.65 (1.56–4.20) | 2.75 (1.61–4.45) |
| Albumin, g/L | 6 (1.5) | 41.6 ± 3.1 | 41.4 ± 3.0 |

Values are mean ± SD or median (IQR). Similar distributions are a plausibility diagnostic and do not by themselves verify the missing-at-random assumption.

Supplementary Table S6. Delta-adjustment sensitivity analysis for departures from missing at random

| Scenario for imputed UACR and CRP values | OR per 10 ng/mL higher 25(OH)D (95% CI) | P value |
| --- | --- | --- |
| Reference missing-at-random model | 0.69 (0.52–0.91) | 0.009 |
| Multiply imputed values ×0.75 | 0.69 (0.52–0.92) | 0.010 |
| Multiply imputed values ×1.25 | 0.70 (0.53–0.92) | 0.011 |
| Multiply imputed values ×1.50 | 0.70 (0.53–0.93) | 0.012 |

Delta adjustments were applied only to imputed UACR and CRP values before refitting extended exploratory Model B. These scenarios do not exhaust plausible nonignorable missingness mechanisms.

Supplementary Table S7. Comparison between photographed patients entering detailed screening and those not taken forward

| Available EMR variable | Entered detailed screening (n = 704) | Not taken forward (n = 423) | SMD |
| --- | --- | --- | --- |
| Age, years | 60.4 ± 8.8 | 58.1 ± 9.2 | 0.26 |
| Male sex, n (%) | 421 (59.8) | 237 (56.0) | 0.08 |
| Inpatient index encounter, n (%) | 445 (63.2) | 164 (38.8) | 0.50 |
| Diabetes duration, years | 11.0 ± 5.3 | 8.7 ± 5.0 | 0.45 |
| Hypertension, n (%) | 451 (64.1) | 236 (55.8) | 0.17 |
| HbA1c, % (n = 675/391) | 7.9 ± 1.4 | 7.6 ± 1.5 | 0.21 |
| eGFR, mL/min/1.73 m² (n = 699/404) | 82.4 ± 18.4 | 88.7 ± 17.6 | 0.35 |
| CKD, n (%) | 102 (14.5) | 38 (9.0) | 0.17 |
| Albumin, g/L (n = 693/398) | 41.5 ± 3.1 | 42.0 ± 3.0 | 0.16 |

Note: Comparisons used only routinely available EMR variables. Conventional independent-group SMDs are descriptive and do not account for selection mechanisms. The greater inpatient proportion and clinical burden among patients entering detailed screening support healthcare selection bias.

Supplementary Table S8. Comparison of selected and non-selected eligible non-DR controls

| Variable | Selected controls (n = 266) | Non-selected eligible controls (n = 170) | SMD |
| --- | --- | --- | --- |
| Age, years | 59.6 ± 8.5 | 62.4 ± 9.0 | 0.32 |
| Diabetes duration, years | 10.1 ± 5.0 | 12.4 ± 5.4 | 0.44 |
| Serum 25(OH)D, ng/mL | 23.4 ± 8.1 | 22.6 ± 8.0 | 0.10 |
| BMI, kg/m² | 25.3 ± 3.3 | 25.5 ± 3.4 | 0.06 |
| HbA1c, % | 7.7 ± 1.3 | 7.9 ± 1.4 | 0.15 |
| SBP, mmHg | 131.3 ± 15.4 | 134.1 ± 15.8 | 0.18 |
| eGFR, mL/min/1.73 m² | 86.7 ± 16.6 | 82.8 ± 18.7 | 0.22 |
| UACR, mg/g | 25.5 (14.5–47.4) | 31.8 (17.2–58.6) | 0.18* |
| CRP, mg/L | 2.3 (1.4–3.7) | 2.6 (1.5–4.2) | 0.13* |

Note: *SMD calculated after log transformation. Deterministic matching altered the age and diabetes-duration profile of selected controls, while the serum 25(OH)D distribution differed only modestly.

Supplementary Table S9. Pre-adjudication inter-rater reliability for diabetic retinopathy assessment

| Pre-adjudication assessment | Categories | Agreement, n/N (%) | Kappa statistic (95% CI) | Weighting |
| --- | --- | --- | --- | --- |
| DR presence | No DR vs any DR | 381/399 (95.5) | κ = 0.90 (0.86–0.94) | Unweighted Cohen κ |
| ICDR severity | No DR to PDR | 348/399 (87.2) | Weighted κ = 0.86 (0.82–0.91) | Quadratic weights |
| Referable DR | No/mild vs moderate or worse | 370/399 (92.7) | κ = 0.84 (0.78–0.89) | Unweighted Cohen κ |

Supplementary Table S10. Concurrent, unadjusted serum 25(OH)D patterns across ICDR severity categories

Note: P for trend used the Jonckheere–Terpstra test for continuous serum 25(OH)D and the Cochran–Armitage trend test for the proportion <20 ng/mL. These descriptive tests were unadjusted and did not account for individual matching.

| ICDR severity category | n | Serum 25(OH)D, ng/mL | 25(OH)D deficiency (<20 ng/mL), n (%) |
| --- | --- | --- | --- |
| No apparent DR | 266 | 23.4 ± 8.1 | 121 (45.5) |
| Mild NPDR | 45 | 20.5 ± 7.2 | 25 (55.6) |
| Moderate NPDR | 49 | 18.7 ± 7.0 | 31 (63.3) |
| Severe NPDR | 23 | 16.9 ± 6.5 | 18 (78.3) |
| PDR | 16 | 15.2 ± 6.0 | 12 (75.0) |
| P for trend | — | <0.001 | 0.002 |

Supplementary Table S11. Season- and medication-adjusted sensitivity models

| Model | Additional specification beyond Extended Model B | n | Matched sets | OR per 10 ng/mL increase (95% CI) | P value |
| --- | --- | --- | --- | --- | --- |
| Extended Model B | Season included | 399 | 133 | 0.69 (0.52–0.91) | 0.009 |
| Sensitivity Model A | Month of blood collection instead of season | 399 | 133 | 0.70 (0.53–0.93) | 0.013 |
| Sensitivity Model B | Insulin use and oral hypoglycemic agents | 399 | 133 | 0.71 (0.53–0.95) | 0.020 |
| Sensitivity Model C | Antihypertensive drugs, RAS blockers, statins, and other lipid-lowering therapy | 399 | 133 | 0.70 (0.52–0.94) | 0.017 |
| Sensitivity Model D | All medication variables listed in Supplementary Table S12 | 399 | 133 | 0.72 (0.53–0.98) | 0.036 |

All sensitivity models were fitted in the multiply imputed full matched sample of 399 participants in 133 matched sets, using Extended Model B as the base specification. Missing covariates were imputed as described in Supplementary Table S4. Model A replaced season with month of blood collection. Model B additionally adjusted for insulin, metformin, sulfonylurea or glinide, DPP-4 inhibitor, GLP-1 receptor agonist, and SGLT2 inhibitor. Model C additionally adjusted for antihypertensive drugs, renin–angiotensin system blockers, statins, and other lipid-lowering therapy. Model D included all medication variables listed in Supplementary Table S12. Because prescription records did not capture adherence, dose, duration, treatment intensity, recent changes, or non-prescription products, these analyses were exploratory and their attenuated estimates were interpreted as broadly similar rather than confirmatory.

Supplementary Table S12. Medication use by diabetic retinopathy status

| Medication variable | DR (n = 133) | Non-DR (n = 266) | SMD |
| --- | --- | --- | --- |
| Insulin use | 71 (53.4) | 106 (39.8) | 0.27 |
| Metformin | 74 (55.6) | 159 (59.8) | 0.08 |
| Sulfonylurea or glinide | 42 (31.6) | 74 (27.8) | 0.08 |
| DPP-4 inhibitor | 35 (26.3) | 60 (22.6) | 0.09 |
| GLP-1 receptor agonist | 10 (7.5) | 25 (9.4) | 0.07 |
| SGLT2 inhibitor | 20 (15.0) | 51 (19.2) | 0.11 |
| Any antihypertensive drug | 89 (66.9) | 155 (58.3) | 0.18 |
| Renin–angiotensin system blocker | 75 (56.4) | 124 (46.6) | 0.20 |
| Statin use | 80 (60.2) | 139 (52.3) | 0.16 |
| Other lipid-lowering therapy | 21 (15.8) | 32 (12.0) | 0.11 |

Notes: Medication use was defined as an active institutional prescription or documented regular use at the index date. Absence was coded as no documented use rather than confirmed nonuse; outside prescriptions and incomplete medication reconciliation remain possible.

Supplementary Table S13. Exploratory quartile-based analysis of serum 25(OH)D

*Trend was modeled by assigning each participant the median serum 25(OH)D value of the participant's quartile. Quartile cut points were sample-derived and were not interpreted as clinical thresholds.

| Serum 25(OH)D quartile | Range, ng/mL | Quartile median, ng/mL | DR / Non-DR, n | Informative sets | Extended exploratory Model B OR (95% CI) | P value |
| --- | --- | --- | --- | --- | --- | --- |
| Q4, highest | ≥28.0 | 32.6 | 16 / 84 | 108 | 1.00 (ref) | — |
| Q3 | 22.0 to <28.0 | 24.7 | 28 / 71 | 108 | 1.45 (0.72–2.92) | 0.300 |
| Q2 | 17.0 to <22.0 | 19.4 | 36 / 64 | 108 | 1.82 (0.92–3.58) | 0.084 |
| Q1, lowest | <17.0 | 13.8 | 53 / 47 | 108 | 2.63 (1.32–5.23) | 0.006 |
| P for trend* | — | — | — | 108 | — | 0.003 |

Supplementary Figure S1. Conceptual framework for concurrent association and covariate interpretation

Background and shared clinical factors may influence both serum 25(OH)D and prevalent DR. The line between exposure and outcome denotes the observed concurrent association and does not specify temporal or causal direction. Added biomarker domains are shown separately because their roles as shared causes, disease-severity markers, mediators, or colliders cannot be distinguished in this retrospective cross-sectional analysis. The framework is interpretive and does not define a proven sufficient causal adjustment set.

Supplementary Figure S2. Representative multiple-imputation diagnostics for UACR and CRP

Panels a and c compare observed and pooled imputed distributions on log1p scales. Panels b and d show representative chain means over 20 iterations; the vertical dotted line marks iteration 10. Trace stabilization and distributional similarity support numerical convergence but do not prove the missing-at-random assumption.

Supplementary Figure S3. Restricted cubic spline for serum 25(OH)D and prevalence odds of diabetic retinopathy

Conditional logistic regression used four knots at the 5th, 35th, 65th, and 95th percentiles and the sample median (22.0 ng/mL) as reference. The shaded band is the 95% CI; the rug plot shows the observed 25(OH)D distribution (n = 399). Overall association P = 0.008; P for nonlinearity = 0.214. Tail estimates are supported by fewer observations and should be interpreted cautiously.
